# Circulating ILC precursors expressing CD62L exhibit a type 2 signature distinctly decreased in psoriatic patients

**DOI:** 10.1002/eji.202048893

**Published:** 2021-04-07

**Authors:** Stefania Campana, Claudia De Pasquale, Chiara Barberi, Daniela Oliveri, Giacomo Sidoti Migliore, Bruno Galletti, Claudio Guarneri, Serafinella Patrizia Cannavò, Guido Ferlazzo

**Affiliations:** ^1^ Department of Human Pathology Laboratory of Immunology and Biotherapy, University of Messina Messina Italy; ^2^ Department of Experimental Medicine (DIMES) University of Genoa Genoa Italy; ^3^ Cell Factory Center and Division of Clinical Pathology University Hospital Policlinico G. Martino Messina Italy; ^4^ Unit of Otorhinolaryngology University Hospital Policlinico G. Martino Messina Italy; ^5^ Unit of Dermatology University Hospital Policlinico G. Martino Messina Italy

**Keywords:** ILC2, ILC Precursors, CD62L, Psoriasis

## Abstract

Human CD117^+^CRTH2^neg^ innate lymphoid cells (ILC) comprise multipotent precursors (ILCp), which are able to differentiate into subtypes in response to different signals received in peripheral tissues. NKp46^+^ ILCp have been reported to associate with ILC3 whereas KLRG1^+^ILCp with ILC2, although the latter can also generate other ILC subsets, thus, maintaining a substantial plasticity. We here showed that CD62L is expressed by ILCp exclusively within KLRG1^+^ population and its expression marks a loss of their broad differentiation potential.

Analysis of cytokine production and relevant markers demonstrated that CD62L^+^ILCp mainly differentiate into ILC2 whereas CD62L^neg^ counterpart can also differentiate into other ILC subsets depending on the signals they receive. Remarkably, in peripheral blood of psoriatic patients, where ILC3 are usually enriched, CD62L^+^ILC were drastically reduced, whereas CD62L^neg^ILC2 upregulated both RORγt and NKp46, thus, suggesting an ongoing conversion to ILC3. Therefore, CD62L now emerges as a potential marker to identify a skewing toward type 2 among ILCp.

## Graphical abstract

CD62L expression identifies a population of ILCp more biased toward type 2 profile. Circulating ILCp expressing CD62L are significantly reduced in psoriatic patients resulting in a skewing toward ILC3.

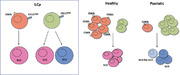

## Introduction

Innate lymphoid cells (ILC) represent an expanding family of innate effector cells that play critical roles in the generation and maintenance of mucosal immunity. ILC lack adaptive antigen receptors and promptly react to signals providing an immediate source of cytokines before the generation of antigen‐specific Th cells, which conversely requires several days. Three major groups of helper ILC are distinguished based on the signature cytokines they produce and the expression of key transcription factors: T‐bet^+^ ILC1 produce interferon‐γ (IFN‐γ), GATA binding protein 3

(GATA3^+^) ILC2 secrete interleukin‐5 (IL‐5), IL‐9, IL‐13, and amphiregulin, and ROR‐related orphan receptor gamma (RORγt^+^) ILC3 mainly produce IL‐22 and IL‐17, thus mirroring, respectively, Th1, Th2, and Th22. Since their discovery, additional subsets of ILC have been reported, although some of them share phenotypic and functional features with ILC subsets initially described, thus probably reflecting, rather than a real distinct lineage, an adaptation of ILC to microenvironment cues, known as functional plasticity. In this regard, human ILC3 in the presence of IL‐12 can convert into ILC1‐like cells and produce IFN‐γ when cultured with IL‐2 or IL‐15 [1]. On the other hand, IL‐23 can play a role in reverting this program and promoting conversion of ILC1 into ILC3 [1]. Increasing reports evidenced that ILC2 also display a certain degree of functional plasticity. Stimulation of ILC2 with Notch ligands induced RORγt expression and IL‐17 production, thus, converting ILC2 into cells with an ILC3‐like profile [2]. Also, both human and mouse ILC2 can convert into ILC1‐like cells that produce IFN‐γ in response to IL‐12 and IL‐1β [3]. More recently, a subset of ILC specialized in the production of IL‐10 has also been identified, although it appears largely related to ILC2 rather than representing a distinct subset [4, 5]. Considering this plasticity, it is becoming evident that ILC functional programs may display a high level of complexity during immune responses, both in healthy homeostasis and disease. The recent discovery of circulating multipotent ILC precursors (ILCp), expressing CD117 but lacking mature “lineage” markers, confirms this complexity. A detailed analysis of ILCp has just started to be unraveled, identifying a heterogeneous population that comprise both NKp46‐expressing ILC, related to IL‐22–producing ILC3, and KLRG1‐expressing ILC that are associated with ILC2. However, the latter ILCp subset seems to represent a transitional stage not yet fully committed to ILC2 fate, since it maintains a broad differentiation potential [6]. In addition, ILC2 include both CD117^neg^ and CD117^+^ cells and the latter exhibits an ILC3‐like signature, thus, revealing a wide heterogeneity. Here, we show that within both ILCp and ILC2 populations, CD62L can discriminate a population more biased to type 2 program.

## Results and discussion

**Figure 1:**
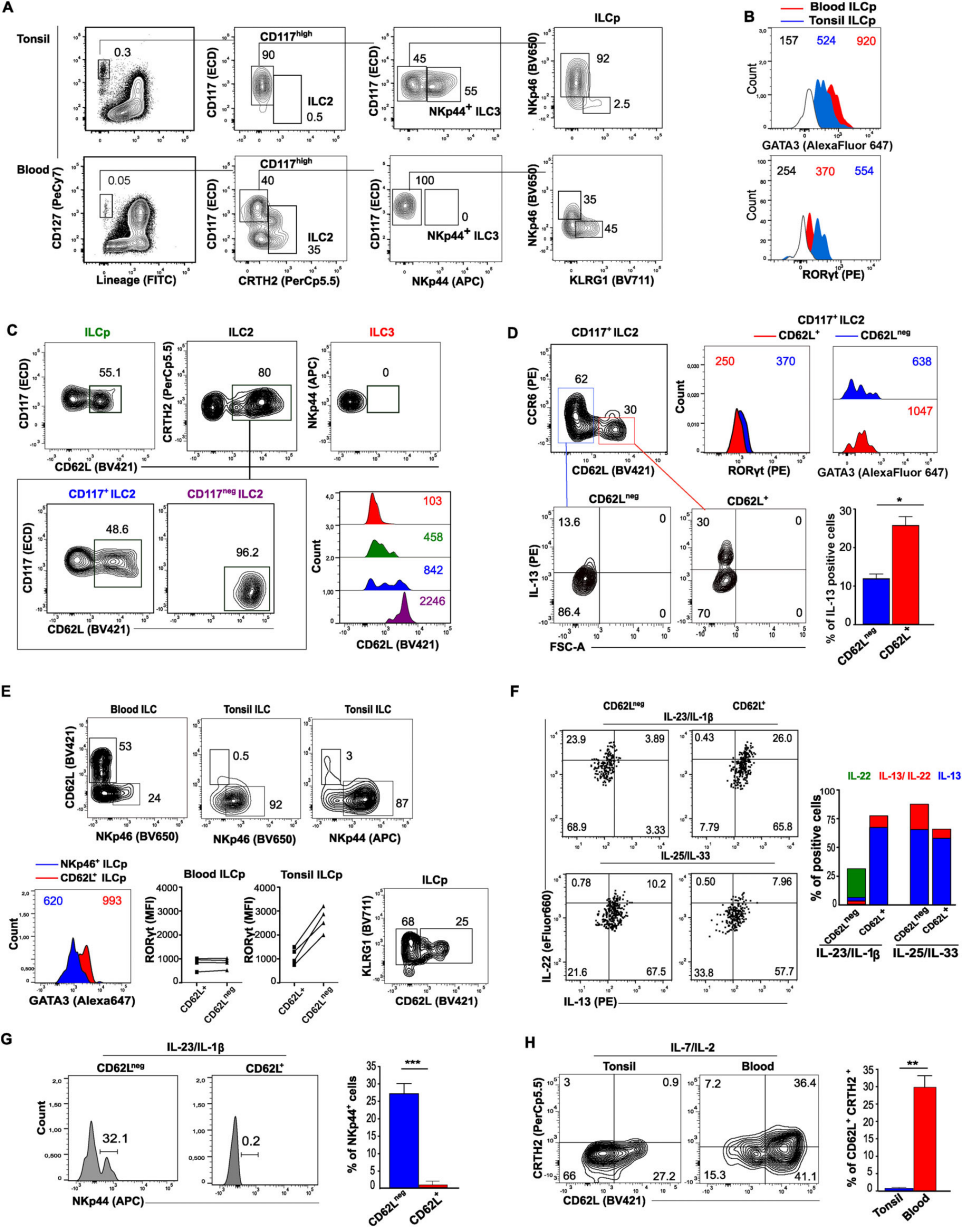
**CD62L identifies a subset of ILCp with type 2 fate**. Analysis of phenotype and cytokine profile of human tonsil and blood‐derived ILCp according to CD62L expression. (**A**) Representative gating strategy used for flow cytometric analysis of ILC subsets in PB and tonsil. (**B**) Comparative analysis of GATA3 and RORγt expression in PB and tonsil ILCp analysed according to the full gating strategy outlined in Supporting Information Fig. S1. Data are representative of experiments performed with six PB and six tonsil donors. (**C**) Analysis of CD62L expression patterns in ILCp, ILC2, and both CD117^+^ and CD117^neg^ ILC2 subsets in PB or ILC3 in tonsil. Data are representative of experiments performed in five PB and five tonsil donors. (**D**) *Upper panels*: analysis of CCR6 versus CD62Lexpression and RORγt and GATA3 was assessed on PB CD117^+^ ILC2 population; data are representative of six independent experiments from six different donors. *Lower panels*: IL‐13 production by both CD62L^+^ and CD62L^neg^ CD117^+^ ILC2 isolated from PB. Data shown are representative of six independent experiments from six donors; bars represent mean values ± SEM of positive cells (**p > 0.01); student's paired *t*‐test. (**E**) *Upper panels*: analysis of CD62L distribution in ILCs from PB and tonsil based on the expression of NKp46 and NKp44 receptors; data are representative of three independent experiments performed on three PB and three tonsil donors. *Lower panels*: GATA3 expression was analyzed in PB NKp46^+^ and CD62L^+^ ILCp (*left panel*); data shown are representative of three experiments from three different donors. RORγt expression was assessed in CD62L^+^ and CD62L^neg^ ILCp from four PB and four tonsil donors (*middle panels*). KLRG1 versus CD62L expression in PB total ILCp (*right panel*); data shown are representative of five independent experiments from five PB donors. (**F**) IL‐22 and IL‐13 production was assessed by intracellular staining on CD62L^+^ and CD62L^neg^ ILCp isolated from PB, cultured with IL‐23/IL‐1β or IL‐25/IL‐33 for 7 days and then stimulated for additional 3 h with PMA/Ionomycine. Data shown are representative of three experiments with three different PB donors. Bars represent mean percentage ± SEM of cytokine producing cells obtained in the three experiments. (**G**) Percentage of cells expressing NKp44 in CD62L^+^ and CD62L^neg^ ILCp isolated from PB and cultured in the presence of IL‐7, IL‐1β, and IL‐23 for 5 days. Data are representative of six experiments with six PB donors; bars represent mean values ± SEM of the percentage of NKP44^+^ cells obtained in the three experiments (*** *p* < 0.001) student's paired *t*‐test. (**H**) Expression of CD62L and CRTH2 in both PB and tonsil ILCp cultured in the presence IL‐7 and IL‐2 for 5 days. Data are representative of seven experiments performed in seven PB and seven tonsil donors. Bars represent mean values ± SEM of the percentage of positive cells obtained in the seven experiments (** *p* < 0.01) student's paired *t*‐test. The donor numbers indicated correspond to the number of experiments performed. Numbers within dot plots represent percentage of cells in the indicated gates/quadrants; numbers within flow cytometry histograms indicate mean fluorescence intensity (MFI) of associated markers.

Human CD117^high^ ILC population, expressing CD127 but lacking CRTH2 and NKp44, have been reported to represent multipotent precursors able to generate all ILC subsets [7]. The new model proposed for ILC differentiation, named “ILC‐poiesis,” suggests that human ILCp systematically circulate and, once in the tissues, progressively differentiate to more committed ILCp [8]. Composition of ILC subsets should consequently be tissue‐specific and, as such, may undergo shifts in pathological conditions, for instance, during tissue inflammation. Therefore, it is conceivable that ILCp become committed in response to cues specific of definite compartments and, during disease, this commitment signals might turn abnormal in damaged tissues. For this reason, besides studying plasticity of mature ILC subsets, it seems useful to also analyse plasticity of ILCp in both normal and altered tissues in order to achieve a more complete picture of the possible spectrum of ILC polarization. In line with these considerations, ILC3 are enriched in inflamed tonsil while ILC2 dominated in peripheral blood (PB) during homeostasis [9] (Fig. 1A). Accordingly, CD117^high^ ILCp showed a different subset distribution in blood and inflamed tonsils, since the frequency of KLRG1^+^ILCp was higher in PB than in tonsil. (Fig. 1A). Analysis of transcription factor expression in these two ILC populations showed that while blood ILCp expressed GATA3, its level is lower in tonsillar counterpart. On the contrary, ILCp in inflamed tonsils expressed much higher amount of RORγt (Fig. 1B). These data reflect the higher frequency of respectively ILC2 in blood and ILC3 in tonsil, supporting the idea that a general commitment toward ILC2 is present in blood, whereas the inflamed microenvironment of resected tonsils can lead to a shift toward ILC3. Although ILCp population can clearly be segregated by the expression of KLRG1 and NKp46 (Fig. 1A), it has been reported that KLRG1^+^ ILCp subset represents a transitional stage biased but not yet fully committed to ILC2 fate. Analyzing the expression of CD62L in different subsets of circulating ILC, we observed that a significant fraction of ILCp expressed this homing marker,:ILC2 showed a high frequency of CD62L^+^ cells and ILC3 did not express it (Fig. 1C). ILC2 are defined by the expression of CRTH2 that includes cells expressing CD117 at different levels [10]. Indeed, CD117 expression distinguishes two functionally distinct ILC2 subsets: CD117^+^ ILC2 is a more plastic subpopulation that expresses RORγt and is able to convert into an ILC3‐like profile, whereas the CD117^neg^ ILC2 display greater potential to produce type 2 cytokines and may represent a fully mature and lineage‐committed ILC2 subpopulation [10]. In view of this consideration, we evaluated CD62L expression in both ILC2 subsets and we found that while CD117^+^ ILC2 expressed only in part CD62L, fully mature CD117^neg^ ILC2 were homogeneously CD62L^+^ (Fig. 1C). Moreover, dissecting CD117^+^ ILC2 according to CD62L expression, we found that CD62L^neg^ fraction exhibited an ILC3‐like signature since it expressed CCR6 and RORγt (Fig. 1D) while the CD62L^+^ counterpart expressed higher level of GATA3 and is endowed with the capability to produce IL‐13 upon stimulation (Fig. 1D). As a whole, these data suggest that CD62L expression is associated to an ILC2 phenotype (Fig. 1C) and that, accordingly, CD62L might discriminate a population of ILCp committed toward ILC2. CD62L^+^ ILCp were present exclusively in the NKp46^neg^/NKp44^neg^ population and they were barely detectable in tonsils, in agreement with the low frequency of ILC2 in these compartments (Fig. 1E). PB CD62L^+^ ILCp showed higher level of GATA3 if compared to NKp46^+^ ILCp (Fig. 1E), and CD62L^+^ in tonsil expressed lower levels of RORγt as compared with the CD62L^neg^ ILC3. CD62L^+^ cells were confined to KLRG1^+^ population, which predominantly generate ILC2 (Fig. 1E). Interestingly, KLRG1^+^ expressing ILCp contain both CD62L^+^ and CD62L^neg^ fractions, raising the question whether CD62L expression might mark a population more biased to type 2 fate.

To address this issue, we evaluated the functional capacities of both CD62L^+^ ILC and CD62L^neg^ ILC by isolating these ILC subsets from PB and culturing them for 7 days in the presence of inflammatory cytokines. We observed that CD62L^+^ILCp produced IL‐13 not only upon culture in the presence of IL‐25 and IL‐33 but also in response to IL‐1β and IL‐23. On the contrary, CD62L^neg^ ILCp could secret IL‐22 upon IL‐1β and IL‐23 stimulation but acquired the capacity to produce IL‐13 in the presence of IL‐25 and IL‐33, suggesting that they can develop into multiple ILC subsets depending on the signals they received (Fig. 1F). Remarkably, when these two ILCp populations were analyzed for the expression of NKp44, a prototypical ILC3‐associated marker, only the CD62L^neg^ population acquired NKp44 while CD62L^+^ counterpart did not (Fig. 1G). These findings support the hypothesis that, upon the acquisition of CD62L, KLRG1^+^ILCp lose their plasticity and become more prone to an ILC2 fate. In agreement with our data, a recent study showed that PB ILCp expressed high level of CD62L and were strong producers of IL‐13 while they did not release IL‐22 following *ex vivo* culture in IL‐1β and IL‐23 for 3 days. [11]. However, in this previous study, all ILCp can produce IL‐13 regardless of culture conditions, while our current results show that CD62L^neg^ ILCp can produce IL‐13 only under specific type 2 stimuli. This apparent discrepancy could rely on differences in experimental methodology employed such as timing of ILCp culture in the presence of inflammatory cytokines.

In light of these new evidences on CD62L expression in ILC, we finally evaluated the capability of ILCp isolated from blood and tonsil to acquire CD62L under unbiased condition (i.e. IL‐7 and IL‐2). We observed that ILCp from blood became almost homogeneously CD62L^+^ and a fraction of them acquired CRTH2, while tonsil counterpart showed only a partial acquisition of CD62L and very low level of CRTH2 expression (Fig. 1H). Therefore, although ILCp show multipotent properties in both tonsil and blood, clear differences exist in the two compartments regarding the proportions of ILCp committed to specific ILC subsets, most likely depending on the environmental signals they receive.

**Figure 2:**
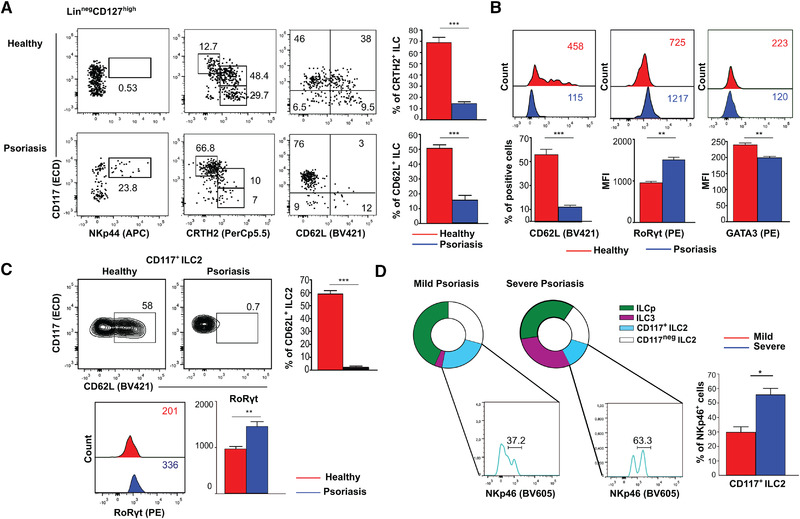
**ILC in psoriatic patients downregulate CD62L expression while acquiring ILC3 signature** Analysis of CD62L expression in peripheral blood ILCp derived from psoriatic patients. (**A**) Frequency of ILC3 and ILC2 in PB of healthy donors or patients with active psoriasis after gating on Lineage^neg^ CD127^high^ cells (*left and middle panels*); CD62L expression on total ILC of healthy donors and patients with active psoriasis (*right panels*). Data are representative of seven experiments performed with seven PB healthy donors and seven PB psoriatic patients. Bars represent mean values ± SEM of the frequency of CRTH2^+^ and CD62L^+^ ILC obtained in all experiments (*** *p* < 0.001) student's paired *t*‐test. (**B**) CD62L, RORγt, and GATA‐3 expression in PB ILCp of healthy donors and psoriatic patients. Data are representative of five experiments performed in PB of five healthy donors and five psoriatic patients. Bars represent mean values ± SEM of MFI of the indicated molecules in the five experiments performed; (**p < 0.01; ***p < 0.001) student's paired *t*‐test. (**C**) CD62L and RORγt expression in PB CD117^+^ILC2 from psoriatic patients or healthy donors. Data are representative of five experiments performed with PB from five healthy donors and five psoriatic patients; bars represent mean values ± SEM of either cell percentage or MFI of the indicated molecules (**p < 0.01; *** *p* < 0.001) Mann–Whitney U test. (**D**) Proportion of ILC subsets among total ILC in the PB of patients with mild (<10 PASI score; 8 patients) or severe (>15 PASI score; 7 patients) psoriasis. A representative staining of NKp46 expression in PB CD117^+^ILC, of both mild and severe psoriasis patients, is shown. Data are representative of stainings obtained in eight mild and seven severe psoriasis patients; bars represent mean values ± SEM of the percentage of NKp46^+^ cells obtained in all patients (*p < 0.05) Mann–Whitney U test. The donor numbers indicated correspond to the number of experiments performed. Numbers within dot plots represent percentage of cells in the indicated gates/quadrants; numbers within flow cytometry histograms indicate mean fluorescence intensity (MFI) of associated markers.

Thus, we analyzed ILCp in PB of psoriatic patients, which harbor, differently from healthy individuals, a significant amount of mature NKp44^+^ILC3, considered new relevant players in the pathogenesis of psoriatic disease [12]. The overall frequencies of ILC in PB of psoriatic patients and healthy donors (HDs) were similar and the increase of ILC3 was accompanied by a drastic reduction of ILC2 percentage and, consistent with our hypothesis, of CD62L^+^CD127^high^ILC (Fig. 2A). ILCp in the PB of psoriatic patients were altered as well, as they did not express CD62L and, at the same time, RORγt expression was increased and GATA3 decreased (Fig. 2B), thus, indicating a switch toward ILC3 profile. Moreover, similarly to tonsil, ILCp in psoriatic patients expressed level of RORγt comparable to NKp44^+^ILC3 (Supporting information Fig. S2). Altogether, this information reveals that the mechanism underlying the relative expansion of a specific ILC subset relies not only in the plasticity of mature ILC or in their selective expansion but also in the different fate of ILCp that might shift according to specific signals.

Remarkably, within CD117^+^ILC2 subset of psoriatic patients, the fraction of CD62L^+^ cells disappeared and the expression of RORγt increased (Fig. 2C). Interestingly, when we compared the composition of ILC subsets in patients with mild (<10 Psoriasis Area Severity Index, PASI, score) and severe disease (>15 PASI score), we found that, in patients with lower PASI score, ILC3 were barely detectable and ILC2 were still well represented, whereas, in severe disease, the frequency of ILC3 cells was significantly higher and ILC2 percentage drastically reduced (Fig. 2D). These data also raised the question whether, in addition to ILCp commitment to ILC3 fate, also mature ILC2 might undergo ILC3 conversion during psoriatic disease. Regarding this issue, in patient's blood a significant fraction of CD117^+^ ILC2 expressed higher levels of both RORγt and NKp46 (Fig. 2C and D) and remarkably, the frequency of NKp46^+^ cells within CD117^+^ILC2 subset increased along disease progression (Fig. 2D). In line with this evidence, a recent study demonstrated that, within skin lesion of patients with psoriasis, the frequency of IL‐17‐producing ILC3 increased at the expense of ILC2 and resulted from a switch of ILC2 to ILC3‐like cells [13]. Noteworthy, ILC2/ILC3 ratio correlates with psoriasis severity and could represent a promising biomarker to assess immunological activity in psoriasis [14]. Since progressive loss of CD62L expression in CD117^+^ILC2 can mark their conversion into ILC3, it might be useful considering the frequency of CD62L^+^ cells among total ILC2 to finely assess disease activity. Altogether, these present results reveal that both ILCp commitment and ILC2 conversion to ILC3 could be mechanisms that occur simultaneously during psoriasis leading to a significant increase of pathogenic ILC3.

In conclusion, the basis of disease‐associated ILC alteration should mainly rely on their plasticity, thus, highlighting the relevance of an early and precise definition of their commitment for therapeutic and/or prognostic purposes. Our current data also indicate that, within KLRG1^+^ILCp, we can take advantage of CD62L assessment to identify a skewing of ILCp toward ILC2 profile. Next, it might be interesting to address whether CD62L expression on ILCp could represent a sensitive early biomarker for type 2‐mediated disease or might correlate with disease activity.

## Concluding remarks

In the last decade, a comprehensive map of human ILC development has been under construction. Lineage^neg^CD127^high^CD117^high^ cells that have been long considered as ILC3, actually represent a novel circulating ILC subset that has the characteristics of human ILCp. Similar to ILC subset distribution, the composition of ILCp could be altered under various disease conditions such as inflammatory diseases and cancer. The current study proposes CD62L as a marker for a better identification of human circulating ILC2p, which can help to investigate ILC development. Also, the identification of early markers defining ILC commitment, coupled with signals driving ILCp differentiation, should be useful for attempting to redirect their effector functions, possibly opening the path to future ILC‐based therapy.

## Materials and methods

### Sample collection

All samples were collected after obtaining informed consent and with approval of protocols by the Ethical Committee of the University Hospital Policlinico *G.Martino*, Messina.

PB was obtained from both HDs and patients affected by active psoriasis. Tonsil tissues were obtained from paediatric tonsillectomies and processed as previously described [15].

### Flow cytometry and gating strategy

The following mAbs were used in this study: anti‐human CD3 (clone HIT3a), CD19 (clone SJ25C1), CD94 (clone HP3D9), CD14 (clone M5E2), CD34 (clone 581), BDCA2 (clone V24‐785), NKp44 (clone p44‐8), NKp46 (clone 9E2), RORγt (clone Q21‐559) and GATA3 (clone L50‐823), CD7 (clone M‐T701), IL‐13 (JES10‐5A2) were all purchased from BD Biosciences; anti‐human CD45 (clone J33), CD45RA (clone 2H4LDH11LDB9) were purchased from Beckman Coulter; anti‐human CD62L (clone DREG‐56), CD127 (clone A019D5), CD117 (clone 104D2), CRTH2 (clone BM16) and KLRG1 (clone 14C2A07), NKp46 (9E2) were purchased from Biolegend; IL‐22 (22URTI) was purchased from ThermoFisher. Intranuclear expression of GATA3 and RORγt was assessed performing fixation/permeabilization procedures according to manufacturer's protocol (Miltenyi Biotec). Total CD127^high^ ILC were gated as lineage (LIN)^neg^ (CD3, CD19, CD94, CD14, CD34, and BDCA2), CD127^high^ cells. Among ILC, ILCp were defined as LIN^neg^ CD127^high^ CRTH2^neg^ CD117^high^ NKp44^neg^, ILC3 were defined as LIN^neg^ CD127^high^ CRTH2^neg^ CD117^+^ NKp44^+^, while ILC2 were defined as LIN^neg^ CD127^high^ CRTH2^+^ CD117^+/neg^ NKp44^neg^. Dead cells were excluded by staining with LIVE/DEAD FIXABLE Aqua Dead dye (Invitrogen). Cells were analyzed according to the full gating strategy outlined in Supporting Information Fig. S1.

Sample acquisition was performed on FACSCantoII or FACSymphony (BD Biosciences) flow cytometers and cell sorting was performed on FACSAria II cell sorter (BD Biosciences). For all flow cytometry and cell sorting experiments, the authors have adhered to the EJI guidelines [16]. Data were acquired by FACS Diva (BD Biosciences) and analyzed by FlowJoVX (Tree Star Inc.) software.

### Cell culture

Freshly isolated fluorescence‐activated cell‐sorted human ILCp were plated at 5 × 10^4^ cells per milliliters in RPMI 1640 plus FBS 10% supplemented with penicillin/streptomycin in 96‐well round‐bottom plates. To evaluate IL‐13 production by CD62L^+^ and CD62L^neg^ CD117^+^ ILC2, freshly CD117^+^ ILC2 were sorted from blood of HDs according to CD62L expression by using FACSAria II cell sorter (BD Biosciences) and stimulated with PMA (10 ng/mL) plus ionomycin (500 ng/mL) for 3 h in the presence of monensin (2 μmol/L; Sigma–Aldrich, St. Louis, MO) and brefeldin (10 μg/mL; Sigma–Aldrich). After 3 h, cells were fixed in 1% paraformaldehyde, permeabilized with saponin 0.1% in PBS, and stained with the PE‐conjugated anti‐IL‐13 antibody IL‐13. To evaluate IL‐22 and IL‐13 production by both CD62L^neg^ and CD62L^+^ ILCp, cells were sorted from blood of HDs according to CD62L expression and cultured for 7 days with human stromal cells [17] in the presence of recombinant IL‐1β/IL‐23 (50 ng/mL, Miltenyi Biotec, each) or IL‐25/IL‐33 (50 ng/mL, Miltenyi Biotec, each). After 7 days, cells were stimulated with PMA (10 ng/mL) plus ionomycin (500 ng/mL) for 3 h in the presence of Monensin (2 μmol/L; Sigma–Aldrich) and Brefeldin (10 μg/ mL; Sigma–Aldrich) and cytokine production was assessed by intracellular staining. To evaluate transdifferentiation of both CD62L^neg^ and CD62L^+^ ILCp into ILC3, cells were sorted as abovementioned and cultured with IL‐7, IL‐1β, and IL‐23 (each at 50 ng/mL, Miltenyi Biotec). After 5 days, cells were harvested and analyzed for the expression of NKp44. To evaluate the capability of tonsil and blood ILCp to acquire CD62L and CRTH2 expression under unbiased condition, ILCp were sorted as LIN^neg^ CD127^high^ CRTH2^neg^ CD117^+^ NKp44^neg^ CD62L^neg^ from both tonsil and blood of HDs and cultured for 5 days in the presence of recombinant IL‐7 (50 ng/mL, Miltenyi Biotec) and IL‐2 (500 UI/mL). After 5 days, cells were analyzed for the expression of CD62L and CRTH2.

### Statistical analysis

Depending on data, a paired Student's *t*‐test, a Mann–Whitney U test were applied to evaluate statistical significance. *p*‐values lower than 0.05 were considered statistically significant (*p < 0.05; **p < 0.01; ***p < 0.001). Statistics were calculated using GraphPad Prism 4 software.

## Conflict of Interest

The authors declare no conflict of interest that pertain to this work.

### Peer review

The peer review history for this article is available at https://publons.com/publon/10.1002/eji.202048893.

**Abbreviations**
ILC: innate lymphoid cells
ILCp: Innate lymphoid cell precursors
GATA3: GATA binding protein 3
KLRG1: Killer cell lectin‐like receptor G1
PB: peripheral blood
RORγt: ROR‐related orphan receptor gamma

## Supporting information

Supporting information

## Data Availability

The data that support the findings of this study are available within the article and its supplementary materials.
