# Supplementary material for: Circulating ILC precursors expressing CD62L exhibit a type 2 signature distinctly decreased in psoriatic patients
Source: Eur J Immunol. 2021 Apr 7;51(7):1792–8. doi: 10.1002/eji.202048893 (PMC8360187; doi:10.1002/eji.202048893)
Supplement: Supplementary file 1 — Supporting information [file EJI-51-1792-s001.pdf]

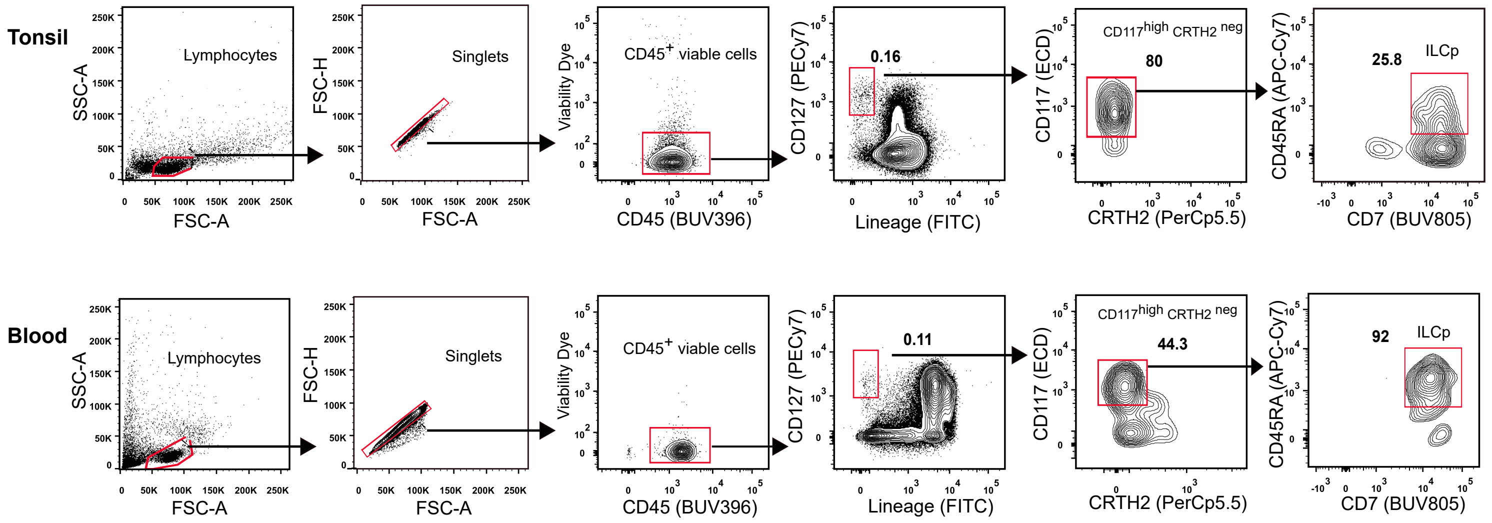

Figure S1. Comprehensive flow cytometry gating strategy employed to identify ILCp population in PB and tonsil.

After gating on lineage negative cells (CD3, CD19, CD56, CD14, BDCA2, CD34), ILCp were defined as $CD127^{+}/CD117^{high}/\ CRTH2^{neg}NKp44^{neg}/\ CD7^{+}/CD45RA^{+}$ cells.

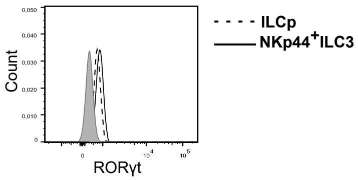

Figure S2. Analysis of RORγt expression on PB ILCp and NKp44^{+}ILC3 from psoriatic patients.
RORγt expression was assessed by flow cytometry on PB ILCp (dotted line) and NKP44^{+}ILC3 (solid line). Grey histogram represents isotype matched irrelevant antibody.
